# Transcriptional Response of Durum Wheat During Interaction with *Debaryomyces hansenii* and *Fusarium graminearum*

**DOI:** 10.3390/ijms27010457

**Published:** 2026-01-01

**Authors:** Wioletta E. Pluskota, Jan P. Jastrzębski, Łukasz Paukszto, Urszula B. Wachowska

**Affiliations:** 1 Department of Plant Physiology, Genetics and Biotechnology, University of Warmia and Mazury in Olsztyn, 10-719 Olsztyn, Poland; 2 Department of Botany and Nature Protection, University of Warmia and Mazury in Olsztyn, 10-721 Olsztyn, Poland; 3 Department of Entomology, Phytopathology and Molecular Diagnostics, University of Warmia and Mazury in Olsztyn, 10-720 Olsztyn, Poland

**Keywords:** biological control, Fusarium head blight (FHB), RT-qPCR, RNA-Seq, *Triticum turgidum* ssp. *durum*

## Abstract

*Debaryomyces hansenii*, a yeast that plays an important role in several agri-food processes, is increasingly being investigated as a biological protection factor against fruit and grain pathogens because of its ability to inhibit the growth of unwanted microorganisms. Biological plant protection can be used as an alternative to pesticides, which cannot be used in organic farming. The mechanism of action associated with the biocontrol capacity of *D. hansenii* against *Fusarium graminearum*, the agent of Fusarium head blight in wheat, and its involvement in induced plant defense were studied in *Triticum turgidum* ssp. *durum* on the mRNA level. A total of 3432 differentially expressed genes (DEGs) of *T. turgidum* ssp. *durum* were identified by using RNA-Seq analysis in a sample primed with *D. hansenii* before pathogen application in comparison to a non-protected sample. Upregulated DEGs encode the proteins involved in cell wall biosynthesis and their modification, photosynthesis, secondary metabolism, and cytoskeleton organization. Among the DEGs, candidate transcription factors as well as protein kinases involved in the signal transduction activated by *D. hansenii* were also shown. Priming durum wheat seedlings with *D. hansenii* leads to enhancing the cell wall structure, which increases plant resistance to fungal infection.

## 1. Introduction

*Triticum turgidum* ssp. *durum* (also known as *T. durum* or durum wheat) is a widely grown crop, a tetraploid (AABB) domesticated species (2*n* = 4x = 28) originating from intergeneric hybridization and polyploidization between *Triticum urartu* (A genome) and *Aegilops speltoides*-related species (B genome), whose grain, owing to its physical and chemical characteristics, is mostly used for making pasta, bulghur, couscous, puddings, flat and leavened bread, and many other Mediterranean traditional foods [1]. Wheat (*Triticum* spp.), one of the most important crops worldwide, is susceptible to a wide range of fungal diseases. Its cropped area amounts to more than 200 million ha worldwide [2], of which around 14 million ha in 2018–2022 was cropped with durum wheat [3]. Fusarium head blight (FHB), also known as head scab, is one of the most destructive diseases of grain crops because it has an adverse impact on both quantity (yield losses) and quality (toxin production). Fusarium head blight, caused by *Fusarium graminearum* species complex (FGSC), is a devastating disease of wheat and barley. The FGSC consists of at least 16 species, morphologically defined as a single panmictic species, *F. graminearum*, which were separated in the first decade of 2000 by employing multilocus sequencing [4,5,6,7,8,9]. *Fusarium graminearum* (teleomorph *Gibberella zeae* Schwabe) is the dominant species of the FGSC studied [4]. Infected cereal kernels, depending on the chemotype of *F. graminearum*, are contaminated with trichothecene mycotoxins. Trichothecenes, that is, deoxynivalenol (DON) and nivalenol (NIV), alongside acetyl derivatives (3-acetyldeoxynivalenol (3ADON), 15-acetyldeoxynivalenol (15ADON), and 4-acetylnivalenol (4ANIV)) and NX toxins, affect the metabolism of plants, animals, and humans by inhibiting the protein biosynthesis process. Although these compounds are not genotoxic in themselves, they activate genotoxic substances present in a diet or, with the participation of the microbiome, can cause DNA damage, leading to neoplastic processes [10]. Another threat to people is posed by modified mycotoxins, including conjugated mycotoxin forms, undetectable with techniques used to detect free mycotoxins produced in plants and animals, for example, deoxynivalenol-3-glucoside (D3G) [11].

Wheat is susceptible to *Fusarium* infection from anthesis to the soft dough stage of kernel development [12]. Resistance to FHB, controlled by quantitative trait loci (QTL), is affected by morphological and developmental features [13,14] as well as the content and composition of cell wall polymers [15,16]. Wheat cultivars with varying resistance against Fusarium head blight display distinct defense strategies. A cultivar resistant to FHB demonstrates a more robust immune response throughout all grain filling stages by upregulating genes that actively destroy pathogens and eliminate toxins. In contrast, a highly susceptible cultivar engages in passive toxin prevention by upregulating genes linked to tyramine biosynthesis early on, a mechanism potentially involved in strengthening the cell wall [17]. Wheat cultivars that were partially resistant to *F. graminearum* showed increased callose deposition in the transition zone of the spikelet’s rachilla and rachis [18].

Durum wheat is more susceptible to Fusarium head blight (FHB) than other classes of wheat [19,20]. The resistance to FHB within different subspecies or lines of *T. turgidum* varies and ranges from moderate to high resistance [21]. The lack of a resistant source, unlike other types of wheat, means that it is impossible to apply a breeding approach to select QTL traits [22,23]. At least 550 QTLs for FHB have been identified in common wheat [24], compared to about a dozen QTLs in durum wheat [19]. Some stable QTLs in durum wheat were associated with morphological characteristics and co-located with the *Rht-B1* gene on chromosome 4B controlling the height of a plant [25] and locus q on chromosome 5A [26]. Introgression of the major FHB-resistance QTL *Qfhb1* (formerly known as *Qfhs.ndsu-3BS* or simply called *Fhb1*) derived from hexaploid wheat reduced FHB severities in diverse durum wheat backgrounds [27,28].

Ding et al. [29] show that infection of Chinese landrace Wangshuibai by *F. graminearum* induces genes associated with signaling pathways mediated by salicylic acid (SA), jasmonic acid (JA), ethylene (ET), calcium ions, phosphatidic acid (PA), as well as reactive oxygen species (ROS) production and scavenging, antimicrobial compounds synthesis, detoxification, and cell wall fortification. JA-responsive genes were found at the late stage of *F. graminearum* infection, while SA-responsive genes were detected at the early stage of infection. Reduction in *F. graminearum* growth and FHB symptoms was observed in wheat heads treated with JA [30]. In contrast, the auxin pathway is associated with susceptibility of wheat cultivars to *F. graminearum* infection. A susceptible genotype of wheat (Robin) displayed higher auxin accumulation during infection relative to a resistant genotype [31].

Triazole fungicides are the most effective strategy to reduce FHB in wheat because most wheat cultivars, especially durum wheat ones, are particularly susceptible to this disease [32]. Unfortunately, in recent years, strains of *F. graminearum* resistant or less vulnerable to some triazole fungicides have been detected on wheat fields in Germany, Lithuania, and Serbia [33,34,35]. Moreover, Directive 128/2009/EC concerning sustainable use of pesticides in Europe imposes the obligation on all EU member states to implement integrated plant management (IPM) since January 2014, with priority given to agrotechnical and biological pest control methods [36]. Hence, the search for biological methods to control FHB seems to be a priority task. The use of the strain *Debaryomyces hansenii*, which is often detected in dairy products, to protect spikes is completely safe [37]. The European Food Safety Authority (EFSA) has classified this species as presenting a qualified presumption of safety [38]. Although *D. hansenii* has proven useful in the protection of stone fruit against *MONILINIA FRUCTIGENA* and *M. fructicola* [39] and common wheat against FHB [40], we still do not know how it affects the wheat’s defense mechanisms during an infection.

We applied RNA sequencing (RNA-Seq) technology to investigate the transcriptomic response of *Triticum turgidum* primed with *D. hansenii* and subsequently inoculated with *F. graminearum*, in order to analyze selected defense mechanisms in durum wheat plants within an antagonist–pathogen–host system. The effect of *D. hansenii* application on the host response was examined at the stage when the first visible symptoms of *F. graminearum* infection usually begin to appear in durum wheat.

## 2. Results

### 2.1. Gene Expression Analysis

Eighty-one percent of the total, 411.8 million, generated RNA-Seq reads passed the quality trimming. Among 332 million trimmed reads, 76% and 78% were aligned to the *T. turgidum* genes (annotation version Svevo.v1) reference genome, respectively, for spikes protected with *D. hansenii* and inoculated with *F. graminearum* and spikes inoculated with *F. graminearum* (Table S1). The data have been deposited in the ENA database at accession number PRJEB55781.

**Figure 1:**
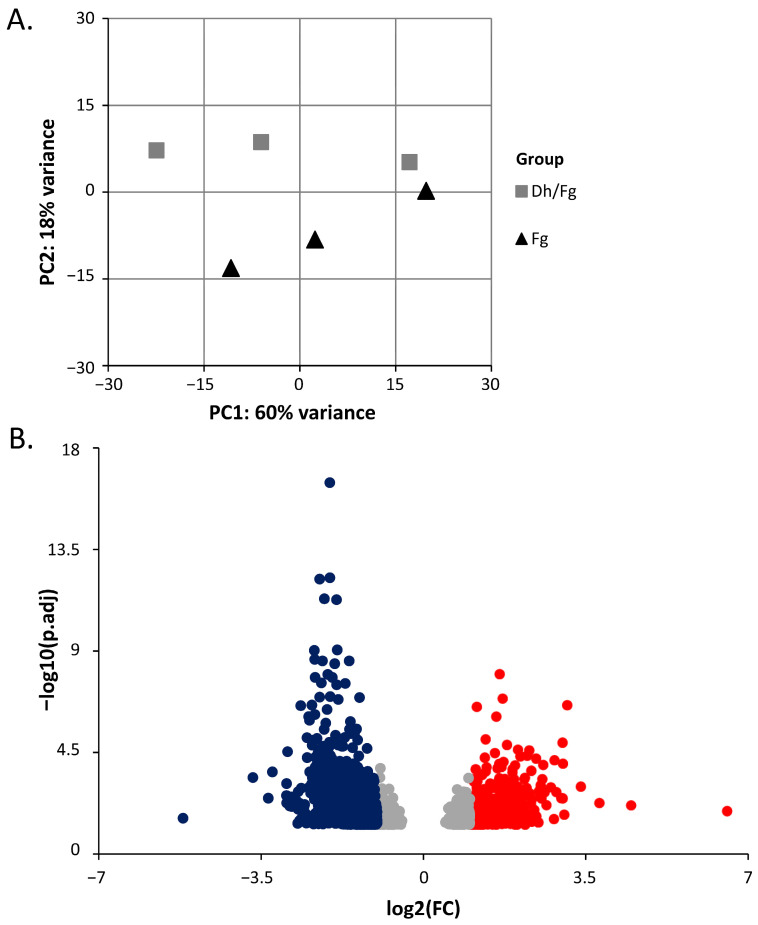
Overview of mRNA transcripts. (**A**) Principal component analysis (PCA) of variance in replicate samples used in the transcriptomic study. The gray square represents *T. turgidum* protected by *D. hansenii* (Dh/Fg), while the black triangle represents non-protected (Fg) samples. (**B**) A volcano plot showing DEGs of *T. turgidum* in two groups with FDR < 0.05 and |log2(FC)| > 1 as the threshold. The red dots represent significantly upregulated genes, the blue dots represent significantly downregulated genes, and the gray dots represent genes that did not meet DEG criteria in the Dh/Fg group relative to their expression in the Fg group.

Principal component analysis (PCA) of the normalized read counts of all samples for the 1000 most variable genes shows that the principal component 2 (PC2) segregates two experimental condition clusters separately. PC2 is responsible for 18% of the variability among samples. The first two principal components (PCs) explained 78% of the variability among samples (Figure 1A, Table S2).

In total, 4354 mRNA transcripts of *T. turgidum* protein-coding genes were significantly differentially expressed using a threshold of padj < 0.05. Among those genes, 14% (620 DEGs; log2(FC) ≥ 1) and 65% (2812 DEGs; log2(FC) ≤ −1) were up- or downregulated in samples protected with *D. hansenii* (Figure 1B, Table S3). Approximately twenty-one percent of expressed genes did not meet the criteria for differential expression in the Dh/Fg group relative to the Fg group (922 genes, |log2(FC)| < 1; Table S4).

DEGs were grouped into functionally related gene clusters based on shared annotation terms using the DAVID Gene Functional Classification Tool, which applies kappa statistics to measure the degree of functional annotation overlap and thereby reveal major biological processes and functional pathways underlying the transcriptional responses [41]. Among the upregulated genes, the three most prominent clusters were associated with phenylpropanoid biosynthesis, including genes encoding dirigent proteins; lignin catabolism, represented by genes encoding laccases; and cell wall organization, involving genes such as xyloglucan endotransglucosylase and pectin acetylesterase. In addition, the upregulated DEG clusters included genes encoding nitrogen metabolite repression proteins (NmrA-like), glucosidases, a phytocyanin domain–containing protein, microtubule-associated proteins, expansins, chlorophyll a/b-binding proteins, carboxypeptidases, peroxidases, and Jacalin-type lectin domains. In contrast, the three most prominent clusters of downregulated genes—more numerous than those observed among the upregulated genes—were related to glutathione metabolism, including genes encoding glutathione transferases; membrane components; and serine-type endopeptidase inhibitors. The distinction in cluster numbers highlights a significant difference in the complexity of responses between up- and downregulated genes. The upregulated genes appear to be more focused on specific processes related to cell wall biosynthesis and modification, while the downregulated genes encompass a broader range of cellular functions, as determined by a method that groups genes based on the strength of functional annotation overlap (Table S5).

### 2.2. GO Enrichment and KEGG Pathway Analysis of DEG

**Figure 2:**
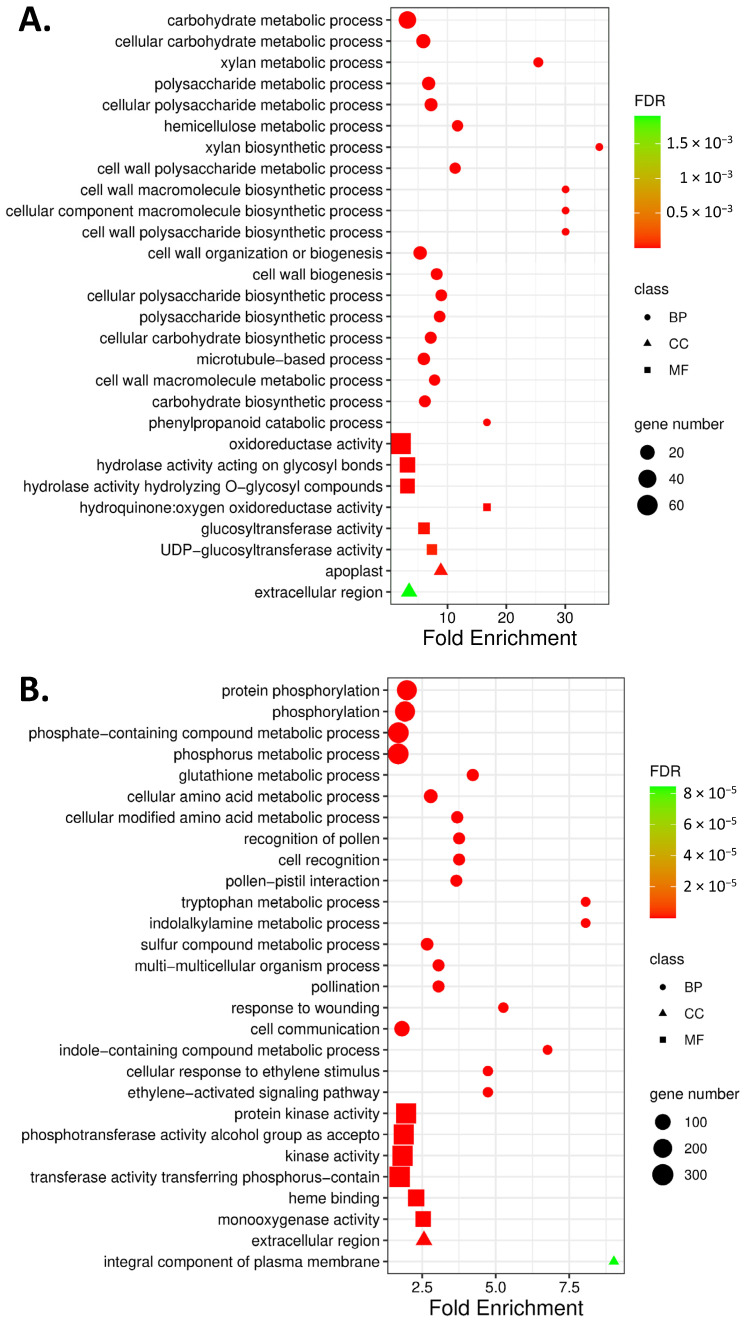
Gene ontology (GO) enrichment analysis of upregulated (**A**) and downregulated (**B**) DEGs in *T. turgidum* spikelets protected with *D. hansenii* in response to *F. graminearum* infection, relative to their expression in non-protected spikelets infected with *F. graminearum*. The top 20 biological processes (BP; marked as wheels), 6 molecular functions (MF; marked as a square), and 2 cellular components (CC; marked as triangles) are shown; point size denotes gene count, color denotes the *p*-value, and the *x*-axis denotes the fold enrichment. For the complete GO list, see Table S6.

In order to classify the function of DEGs, we used GO and KEGG pathway annotation to perform functional enrichment analysis. Gene ontology (GO) enrichment analysis revealed that the effect of the application of *D. hansenii* on the accumulation of *T. turgidum* gene transcripts involved for over 64 biological processes (BP, 24 DEGs upregulated and 40 DEGs downregulated) and over 50 molecular functions (MF, 6 DEGs upregulated and 44 DEGs downregulated) with False Discovery Rate (FDR) of 1 × 10^−4^ applied to filter. BP terms of DEGs differ in the case of up- and downregulated genes (Table S6). Significantly, upregulated genes encode proteins that were localized in the apoplast and extracellular region. Whereas proteins encoded by downregulated genes were located not only in the extracellular region but also represented integral components of the plasma membrane (Figure 2). Plant treatment with *D. hansenii* altered the expression of genes that were mainly involved in cell wall metabolism, secondary metabolic process, and cytoskeleton organization (Figure 2A, Table S6A). Whereas downregulated DEGs were mainly associated with protein modification processes, amino acid metabolic processes, pollination, cell recognition, glutation metabolic processes, biosynthesis and metabolism of tryptophan and indole alkylamines, signal transduction induced by ethylene, and response to wounding (Figure 2B, Table S6B).

**Figure 3:**
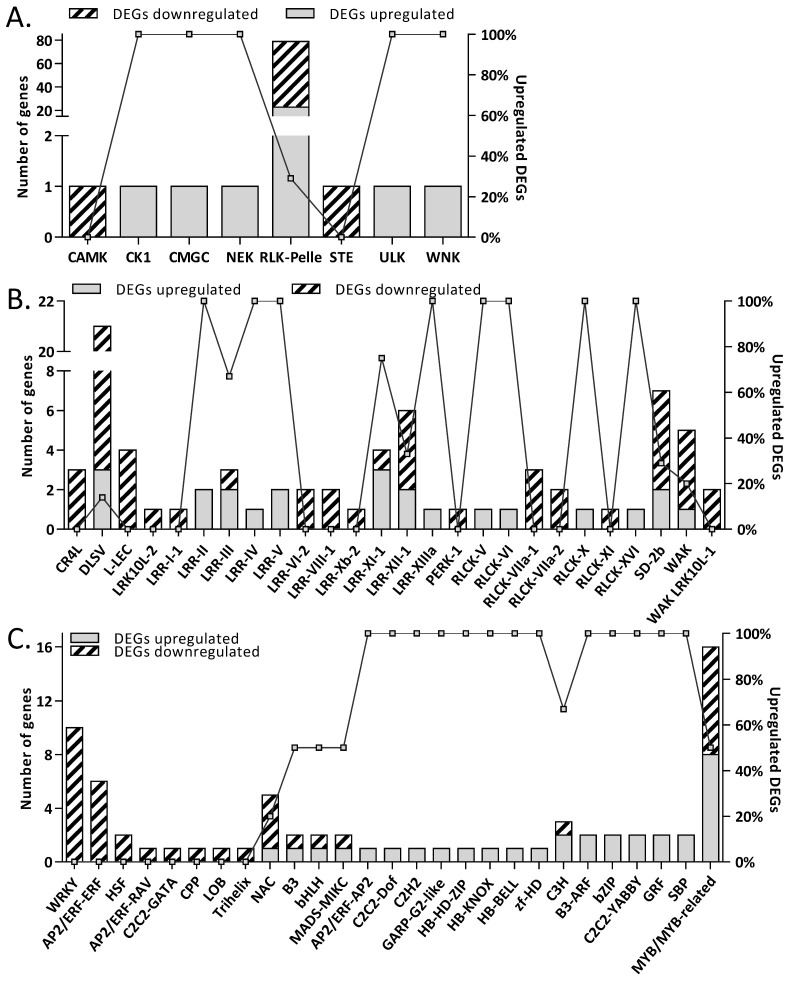
Effect of *D. hansenii* treatment on *T. turgidum* protein kinases (**A**,**B**) and transcription factors (TFs) (**C**) mRNA accumulation. The left *y*-axis shows the number of up- and downregulated DEGs, while the right *y*-axis shows the proportion of upregulated DEGs in each group. Gray-shaded bars and diagonal striped bars indicated the number of up- and downregulated DEGs, respectively. (**A**) Distribution of differentially expressed protein kinase genes classified into major kinase groups. (**B**) Distribution of RLK/Pelle subfamilies. Kinase groups include WNK (with no lysine), ULK, STE, RLK/Pelle (receptor-like kinases), NEK, CMGC, CK1, and CAMK (Ca^2+^/calmodulin-dependent protein kinases). (**C**) Transcription factor (TF) classes showing altered mRNA accumulation in response to *D. hansenii* treatment.

Under molecular function (MF) only two categories, oxidoreductase activity (GO:0016491) and hydroquinone:oxygen oxidoreductase activity (GO:0052716), were common in both up- and downregulated DEGs. 4 (UDP-glucosyltransferase (GO:0035251) and glucosyltransferase activity (GO:0046527) as well hydrolase activity acting on glyco-syl bonds (GO:0016798) and hydrolyzing O-glycosyl compounds (GO:0004553)) and 42 more GO terms were unique, respectively, in up- and downregulated DEGs. Among the downregulated DEGs, the application of *D. hansenii* reduced the accumulation of mRNA protein that binds heme (GO:0020037), iron ion (GO: 0005506), and tetrapyrrole (GO: 0046906). Additionally, MFs of downregulated DEGs were enriched for protein kinase activity (GO:0004672, GO:0016301) as well as DNA-binding transcription factor activity (GO: 0003700) (Table S6). The levels of mRNA transcripts encoding kinases belonging to the three groups (CAMK, RLK-Pelle, and STE) and transcription factors (TF) belonging to the 14 groups were decreased by yeast application (Figure 3A,C). The receptor family RLK-PELLE, comprising approximately 12,000 genes, was the largest group of kinases whose individual members were either activated or downregulated in response to the application of biological protection (Figure 3A). The majority of mRNA transcripts were downregulated in the DLSV subfamily of RLK-PELLE (Figure 3B).

Transcription factors from AP2/ERF-RAV, AP2/ERF-ERF, C2C2-GATA, CPP, HSF, LOB, Trihelix, and WRKY were uniquely downregulated (Figure 3C). In contrast, mRNA transcript levels of some members of B3, bHLH, C3H, MADS-MIKC, MYB, and NAC-related transcription factors were downregulated and upregulated (Figure 3C). The accumulation of TF transcripts that are members of groups such as AP2/ERF-AP2, B3-ARF, bZIP, C2C2-YABBY, C2C2-Dof, C2CH2, GARP-G2-like, GRF, HB-HD-ZIP, HB-KNOX, HB-BELL, SBP, and zf-HD was observed only among upregulated DEGs (Figure 3C). The WRKY, MYB, NAC, and AP2/ERF-ERF were over-represented downregulated TF families. While MYB composed an over-represented upregulated TF family. Moreover, the use of *D. hansenii* leads to changes in the accumulation of mRNA of transcription regulators. In contrast to the abundance of AUX/IAA mRNA, the accumulation of mRNA transcriptional regulators such as TRAF (tumor necrosis factor receptor-associated factors) was reduced.

**Figure 4:**
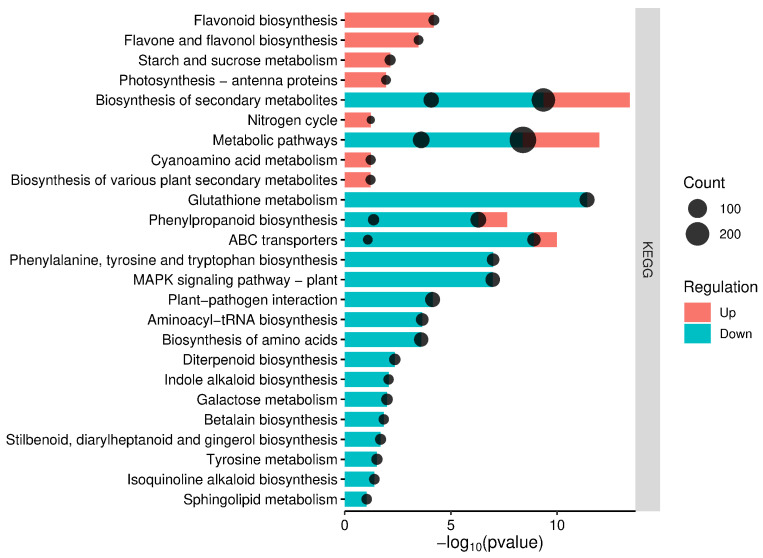
KEGG pathways enriched with orthologs of *T. turgidum* DEGs in bread wheat. The size of each black circle corresponds to the number of DEGs involved in a particular pathway.

In order to identify biological pathways potentially associated with DEGs, we mapped them into the KEGG database and compared the results with the whole transcriptome background. The KEGG analysis was conducted using orthologs of *Triticum turgidum* identified in bread wheat (*Triticum aestivum* L.). Orthologs found in the *T. aestivum* genome represented more than 96% of the DEGs detected in *T. turgidum*. The KEGG analysis revealed 29 pathways associated with the wheat defense response to *Fusarium*, which were altered by protection from *Debaryomyces hansenii* (Figure 4). Both upregulated and downregulated orthologs from *Triticum aestivum* were associated with pathways related to secondary metabolite biosynthesis, general metabolic processes, and phenylpropanoid biosynthesis, with a *p*-value < 0.05. The orthologs of upregulated DEGs from *T. durum* were also associated with specific pathways such as flavonoid biosynthesis, and flavone and flavonol biosynthesis—pathways that contribute to the production of various secondary metabolites. Additionally, some genes were involved in carbohydrate and energy metabolism. The orthologs of downregulated DEGs from *T. durum* participated in glutathione metabolism, membrane transport, amino acid biosynthesis and metabolism, signal transduction, environmental adaptation, and translation. Furthermore, these orthologs were engaged in pathways such as indole alkaloid, betalain, stilbenoid, diarylheptanoid, and gingerol, as well as isoquinoline biosynthesis—processes that facilitate the biosynthesis of secondary metabolites (Figure 4, Table S7).

### 2.3. Quantitative Real-Time Expression Analysis

**Figure 5:**
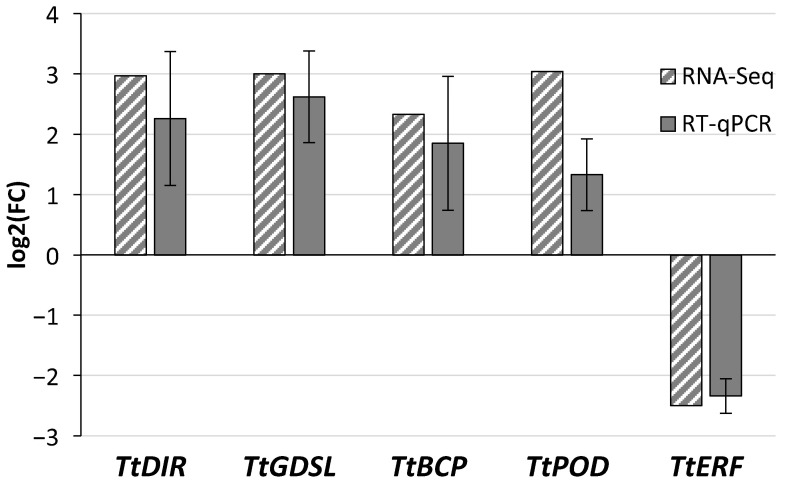
Comparison of the log2(FC) (Dh/Fg vs. Fg) expressed values between the RNA-Seq and RT-qPCR approaches for the 5 target genes chosen for validation. Error bars represent the standard error of the mean.

DEGs encoding proteins involved in cell wall modification (*TtDIR* and *TtGDLS*), ethylene response (*TtERF*), and oxidoreductase activity (*TtPOD* and *TtBCP*) were selected to validate RNA-Seq expression patterns using relative transcript quantification by qPCR. The ethylene-activated signaling pathway was among the biological processes enriched in downregulated DEGs (Figure 2B, Table S6B), whereas oxidoreductase activity was one of the molecular function categories enriched in upregulated DEGs (Figure 2A, Table S6A). The relative expression of *TtDIR* (TRITD2Av1G273910), *TtGDSL* (TRITD7Av1G231170), *TtBCP* (TRITD6Bv1G185120), *TtPOD* (TRITD7Bv1G190550), and *TtERF* (TRITD2Bv1G206010) confirmed the results of the high-throughput analysis. The absolute values of log2(FC) for selected DEGs identified in RNA-Seq analysis ranged from 2 to 3, specifically 2.97, 3.00, 2.33, 3.04, and 2.50, respectively (Table S3). The relative expression of those DEGs, determined by quantitative PCR, followed a similar pattern (Figure 5).

**Figure 6:**
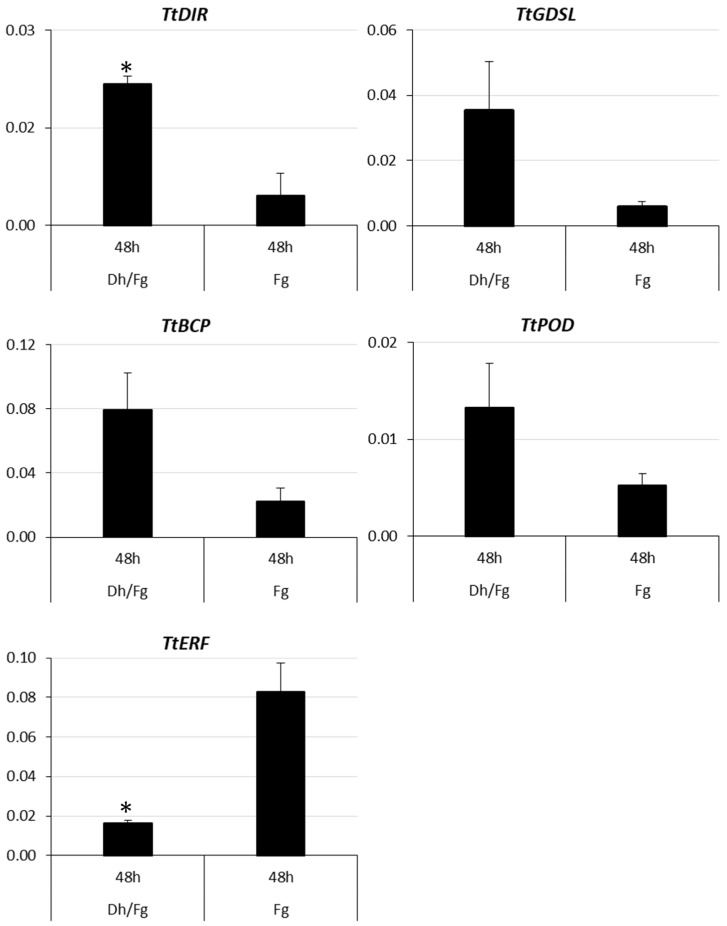
Relative expression of DEGs (*TtDIR*, *TtGDSL*, *TtBCP*, *TtPOD* and *TtERF*) in kernels inoculated only with *F. graminearum* (Fg) and in kernels protected with *D. hansenii* and inoculated with *F. graminearum* (Dh/Fg) in reverse transcription quantitative PCR (RT-qPCR). Asterisk (*) represents a significant difference between groups (*p* < 0.05). Mean and standard error were estimated with data from three independent replicates.

The mean value of *TtDIR*, *TtGDSL*, *TtBCP*, and *TtPOD* expression was increased in contrast to *TtERF*, which was reduced in spikelets protected by *D. hansenii* (Figure 5 and Figure 6).

### 2.4. Expression of D. hansenii Gene

In total, 2029 genes were mapped to the *D. hansenii* CBS767 transcriptome. Detected *D. hansenii* genes were separated into 99 categories based on KEGG methods. Among them, 26 KEGG pathways were significantly represented. At least 50 genes each were expressed from such categories as metabolic pathways, biosynthesis of secondary metabolites, biosynthesis of amino acids, carbon metabolism, oxidative phosphorylation, ribosome, and purine metabolism (Table S8). The expressed secondary metabolism genes encoded enzymes involved in 137 pathways. Glycolysis and gluconeogenesis (00100), pyruvate metabolism (00620), methane metabolism (00680), tryptophan metabolism (00380), pentose phosphate pathway (00030), and purine metabolism (00230) were specially represented pathways among secondary metabolic pathways in which more than 20 unique genes were expressed. On the contrary, pathways such as biotin metabolism were represented by single genes (Table S9). Additionally, GO enrichment of mapped *D. hansenii* genes reveals the accumulation of mRNA transcripts encoding enzymes involved in the fungal biofilm matrix (Figure S1).

Furthermore, among the expressed *D. hansenii* genes, two *D. hansenii* transcripts (DEHA2G11660 and DEHA2B13464) were detected, whose predicted amino acid sequences showed similarity to the amino acid sequence of yeast killer toxins from *Saccharomyces cerevisiae* and protein from *Millerozyma* acacia with similarity to *K. lactis* toxins, 21.4% and 41%, respectively. Both of them show higher identity in amino acid sequences of K2 killer toxin of *S. cerviseae* as well as *M. acacia* protein at the C-end of the protein, 54% and 70%, respectively (Figure S2). The C terminus of the *M. acaciae* protein contained a lysin motif (LysM, PF01476) domain. Whereas, the C terminus of the K2 toxin contained an uncharacterized domain (PF17276).

## 3. Discussion

Treatment of wheat spikelets with a suspension of *D. hansenii* J1 yeast altered the response of *T. turgidum* spp. *durum* at the transcriptome level, induced by the *F. graminearum* infection process following the inoculation of spikelets. The findings reported in this paper are the first report on the transcriptional reprogramming of a plant exposed to the effect of a biological agent, such as *D. hansenii*, in response to the infection with the pathogen. Thus far, biological control of *D. hansenii*, excluding species for which the yeast was applied on harvested fruit, has been described only for plant species like maize [42], muskmelon [43], pine [44], and durum wheat [45,46]. Recently, *D. hansenii* has been applied as a biofertilizer of cucumbers growing in an iron-deficient environment [47]. Moreover, Núñez-Cano et al. [48] demonstrate that *D. hansenii* functions as a plant growth-promoting yeast (PGPY) in rice plants and may serve as a promising biofertilizer for improving rice productivity and nutrient efficiency in calcareous soils.

The research results suggest that an application of *D. hansenii* may alter wheat resistance owing to the accumulation of transcripts encoding cell wall-modifying enzymes, which compose a network of polysaccharides, proteins, and polyphenols, constituting both a physical barrier during a response induced by these pathogens and a site for perception and signaling of the plant’s immunity. Cell wall strengthening might also prevent the spreading of toxins into cells in a wheat cultivar showing moderate resistance to *F. graminearum* during the grain-filling stage [17]. We have found earlier that an application of *D. hansenii* induces a reduction in the severity of FHB symptoms and reduces the content of DON in durum wheat spikes after inoculation with *F. graminearum* [45]. Likewise, Narusaka et al. [49] observed an increase in the resistance of *Arabidopsis thaliana* and *Brassica rapa* to bacterial and fungal infection after an application of a plant activator prepared from yeast cell wall extract. Yaguchi et al. [50] study carried out on rice cell suspension suggests that yeast cell wall extract enhanced resistance against *Arabidopsis* to *Botrytis cinerea* may activate the JA signaling system to induce plant defense. Based on the presented results, we hypothesize that priming plants with *D. hansenii* may influence pathways related to cell wall reinforcement, thereby enhancing the first line of defense previously described in an FHB-resistant wheat cultivar [51]. Furthermore, altered expression of genes encoding glutathione transferase, peroxidases, and enzymes involved in secondary metabolism may indicate defense responses, including not only cell wall reinforcement but also potential pathogen-directed damage.

### 3.1. Impact of Wheat Priming with D. hansenii on the Expression of Genes Involved in Signal Transduction

In contrast to the findings of Sevillano-Caño et al. [47], our results demonstrate that *D. hansenii* treatment decreases the accumulation of ethylene-related gene transcripts in durum wheat, resembling the effects reported for plant growth–promoting microbes [52]. However, unlike bacteria (PGPB) and fungi (PGPF), *D. hansenii* modifies the accumulation of genes encoding Aux/IAA proteins, which are negative regulators of auxin response [53]. Silencing in wheat EIN2, a central regulator of ethylene (ET) signaling, reduced FHB symptoms, implying that ET signaling may promote wheat susceptibility to *F. graminearum* [54]. Likewise, auxin was also implicated in FHB susceptibility of wheat [31]. Knockdown of *TaTIR1*, the auxin receptor gene, increased FHB resistance. Moreover, exogenous application of auxin or IAA enhanced wheat susceptibility to FHB [55]. The response induced by *D. hansenii* most likely involves signal transduction pathways mediated by transcription factors from the MYB and bZIP families, which—together with WRKY and NAC-domain-containing transcription factors—have been associated with susceptibility in wheat species [56]. In our study, transcript levels of TF from the WRKY family, AP2/ERF-ERF, and NAC decreased in samples protected with *D. hansenii*. These TFs are considered activators of the defense response against FHB, with their transcripts accumulating as the infection progresses, starting at 72 h post-infection [57]. Despite numerous research papers describing the mechanism of wheat’s defense response to FHB, our knowledge of the contribution of kinases in signal transduction in response to infections with *Fusarium* spp. is still fragmentary. Receptor-like kinases, which form the largest family of receptors in plants and play an important role in recognizing pathogen-associated molecular patterns and modulating the plant immune responses to invasive fungi, also constituted the largest group of kinases whose transcript changed after *D. hansenii* application in our studies. Gene encoding one of the receptor-like kinases was highlighted as a contributor to basal defense against FHB disease and points to its importance as an upstream component of SA signaling in wheat bread [58]. Recently, Yan et al. [59] have proposed nine wheat RLKs that might be stress response genes under drought or *F. graminearum* treatments.

### 3.2. Impact of Wheat Priming with D. hansenii on the Expression of Genes Involved in Cell Wall Modification

In this study, the application of *D. hansenii* resulted in an increased level of mRNA transcripts encoding proteins involved in lignification, such as dirigent (DIR), laccase (LAC), and blue copper protein (BCP), as well as in adcrustation, represented by GDSL esterase/lipase. Both types of cell wall modification, lignification and adcrustation, require the activity of peroxidases (POD) [60], the transcripts of which were found to have accumulated in samples protected with *D. hansenii*. Dirigent (DIR) proteins, involved in lignin biosynthesis, modulate cell wall metabolism during abiotic and biotic stress exposure [61]. In turn, laccases (LACs) catalyze the oxidative polymerization of monolignols, but can also be engaged in detoxification of DON as well as salicylic acid signaling, dehydration, and low-oxygen stress under *F. graminearum* infection. Prediction of the three-dimensional structure of TaLACs suggests that some of them may serve as potential deoxynivalenol trappers that prevent DON from entering the cell [62]. In our experiment, elevated transcript levels of *TRITD4Bv1G127580* and *TRITD4Av1G043440* genes, which are homologs of the common wheat’s gene *TaLAC58* (TraesCS4B02G208000) encoding laccase, containing a DON binding site in the substrate pocket, binding a lignin monomer, have been observed in the *D. hansenii* protected sample. In addition to LAC, UDP-glucosyltransferase expressed in the *D. hansenii* protected sample may also be involved in DON detoxification. Recently, UDP-glucosyltransferase conferring deoxynivalenol resistance in *Aegilops tauschii* and wheat has been detected [27,28]. Moreover, similarly to our results, strong upregulation of the gene encoding blue copper proteins (plantacyanins), which function as electron transporters during redox processes and are involved in lignin polymerization, was observed during stress and wounding in some FHB-resistant genotypes [56].

In addition to lignin, another cell wall-modifying polymer is suberin, the deposition of which requires the GDSL esterase/lipase [63], whose mRNA transcript level was increased in the *D. hansenii* protected sample in our study. Basheer et al. [64] showed that GDLS, as well as peroxidases and lipid transfer proteins (LTPs), were upregulated in HvMPK3 knockout lines. Barley lines with the HvMPK3 gene knockout (KO), despite lower ROS levels compared to wild-type plants, showed higher root resistance to *F. graminearum* due to the upregulated levels of cysteine proteases, secretory peroxidases, as well as the stiffening of cell walls by suberin deposition, probably constituting a barrier that prevents pathogen invasion into root cells of HvMPK3 KO lines [64]. Eldakak et al. [65] proposed that a GDSL lipase gene (GDSL) is the most likely candidate gene of *Qfhb1*, a major QTL for FHB resistance in wheat. GDSL lipase, which regulates systemic immunity, was absent in susceptible lines [66].

### 3.3. Impact of Wheat Priming with D. hansenii on the Expression of Genes Involved in the Structure of the Cell Wall

The strengthening of the call wall is not the only factor differentiating the resistance of various wheat cultivars to FHB. During wheat infection by *F. graminearum*, fungal hyphae enter the cell by releasing CAZ-enzyme. Susceptibility of the wall to degrading enzymes produced by pathogens during infection can play a role in the outcome of host–pathogen interactions. Resistance to FHB may be affected by the content and composition of cell wall polymers. Lionetti et al. [15] suggest that pectin methylesterase is involved in wheat response to *F. graminearum* due to detected differences in hemicellulose and pectin polymers in the cell wall of spikes of FHB-resistant and susceptible genotypes. In our study, the *D. hansenii* treatment increased the accumulation of mRNA transcripts of genes encoding enzymes mediating the biosynthesis of cell wall polysaccharides, e.g., xyloglucan xylosyltransferase involved in the biosynthesis of xyloglucan, the most abundant hemicellulose in the primary cell wall, as well as cellulose synthase, fasciclin-like arabinogalactan proteins (FLAs), and trichome birefringence-like (TBL) protein. The accumulation of *TBL* mRNA transcript, of which tbr and tbl3 mutants decreased cellulose and altered pectin composition [67], was observed in barley after *F. graminearum* inoculation [68]. Moreover, TBL proteins are responsible for the acetylation of hemicellulose, enabling the formation of cross-links of polysaccharides in the cell wall, as well as acetyl-substituents that inhibit the enzymatic degradation [69]. Whereas FLA proteins, which belong to arabinogalactans, like TBL, are also involved in cellulose synthase [70].

### 3.4. Potential Direct Antagonism of D. hansenii Toward F. graminearum

The literature data indicate that *D. hansenii* J1, as a biocontrol agent, limits the growth of the pathogen in durum wheat, decreases the level of mycotoxins in plants inoculated with *F. graminearum,* and, to a very limited extent, affects the transcript of the pathogen [45]. Like other biocontrol yeasts, *D. hansenii* affects the pathogen by covering the plant tissues and by inhibiting the growth of the pathogen [45,71]; it also competes for nutrients, inhibits the spore germination, produces the volatile organic compounds (VOCs), or excludes enzymes such as β-1, 3-glucanase or protease [71,72]. The formation of a biofilm by *D. hansenii* on the surface of wheat spikelets or maize kernels, documented on electronograms in previous publications [45,71], is in accord with the currently observed expression of genes encoding enzymes involved in the fungal biofilm matrix as well as in gluconeogenesis, a metabolic pathway that, next to glycolysis, plays a key role in the formation of biofilms by *Saccharomyces cerecisiae* [73]. Further, in our study, the expression of genes encoding homologs of killer toxins most probably indicates the ability of the *D. hansenii* J1 strain to create killer proteins. In their research, Czarnecka et al. [74] found that the *D. hansenii* strains KI2a, MI1a, and AII4b, classified by these authors as killer strains, directly affected the pathogen’s cells. The secreted killer proteins are lethal to sensitive cells, like cells of yeasts and fungi (filamentous fungi), e.g., K2 toxin killer from *S. cerecisiae* binds to β-1-6-D-glucan components in the walls of sensitive cells and then disrupts their ion exclusion barrier of the target cell plasma membrane [75], while the killer protein *Kluyveromyces lactis* shows exochitinase activity [76]. Other killer toxins kill their sensitive cells through inhibition of DNA replication, induction of membrane permeability changes, and arrest of the cell cycle in G1 phase [77].

## 4. Materials and Methods

### 4.1. Plant Material, Growth Conditions, and Biological Protection

The spring cultivar of *Triticum turgidum* subsp. *durum* called Durasol, which is susceptible to infections caused by fungi of the genus Fusarium, was grown in pots 25 cm in diameter and 30 cm in height. Wheat seeds (10 per pot) were sown in horticultural soil. Seedlings were grown in a greenhouse at a temperature of 23 °C/19 °C ± 1 °C (day/night), 16/8 h (day/night) photoperiod, and 80% humidity. Seedlings were watered every other day during the entire experiment. Each pot was fertilized three times with NPK fertilizer in a dose of 2 g (N/P2O5/K2O 13.6/6.4/19.1%; Azofoska, Góra Kalwaria, Poland). Corbel^®^ 750EC fungicide (fenpropimorph; BASF, Warsaw, Poland) was applied in the tillering stage (BBCH 21; [78]) and the stem elongation stage (BBCH 31) to protect leaves against infections caused by *Blumeria graminis* ssp. *tritici*. Each plant, after the tillering phase (BBCH 21; [78]), produced two or three ears, resulting in approximately 25 ears per pot. The experiment had a completely randomized design with three replications.

The culture of *D. hansenii* J1 strain (KX444668) was prepared according to Wachowska et al. [45]. Yeast cells, cultured for four days on potato-dextrose agar (PDA) in the dark at a temperature of 27 °C (Pol-Eco, Wodzisław Śląski, Poland), were suspended in sterile water. At the beginning of flowering (BBCH 61), 10 mL of the suspension with the concentration 8 × 10^6^ cells in 1 mL^3^ supplemented with 0.002% Tween^®^40 (Merck, Darmstadt, Germany) was applied to durum wheat spikes in each pot with a 1000 mL manual sprayer (Marolex, Łomna, Poland). The density of the yeast suspension was determined under an optical microscope (Nikon Eclipse E200; Nikon Europe B.V., Amstelveen, The Netherlands) with a Thoma 50 counting chamber (Marienfeld, Lauda-Königshofen, Germany).

### 4.2. Inoculation of Wheat Spike with Fusarium graminearum

A conidial suspension of the *F. graminearum* F3 strain, a genotype of the DON-producing deposited in GenBank under accession number MZ827461, was cultured on liquid carboxymethylcellulose (CMC) medium [45]. Spores were isolated from 7-day-old *F. graminearum* colonies growing on PDA. 200 µL of the suspension containing 10^6^ spores in 1 mL was added to the liquid CMC medium. The flasks were shaken on a shaking table (120 rpm; DLab, Walnut, CA, USA) in the dark at a temperature of 27 °C (Pol-Eco, Wodzisław Śląski, Poland) for 4 days to promote sporulation. The spore suspension was rinsed with sterile water, centrifuged twice (4500 rpm, 10 min; Eppendorf, Warsaw, Poland), and the liquid medium was removed. Pure spores were diluted in sterile water to a concentration of 16 × 10^6^ in 1 mL. The density of the spore suspension was determined under an optical microscope (Nikon Eclipse E200, Nikon Europe B.V., Amstelveen, The Netherlands) with a Thoma 50 counting chamber (Marienfeld, Lauda-Königshofen, Germany).

At the beginning of the flowering stage (BBCH 61), all spikelets per pot were inoculated with the *F. graminearum* F3 strain 24 h after the application of the *D. hansenii* suspension. Using a micropipette (Ovation; VistaLab Technologies, Patterson, NY, USA), 10 µL of the *F. graminearum* spore suspension was introduced between the lemma and the palea at the base of the stamen. After spike inoculation with *F. graminearum*, durum wheat plants were automatically sprayed with water every 30 min during 16 h of daylight. High humidity was maintained during the first four days after inoculation. A total of 50 inoculated spikelets from five spikes were sampled per biological replicate, with three replicates for each of two treatments: (1) wheat plants inoculated with *F. graminearum* and (2) wheat plants protected with *D. hansenii* and subsequently inoculated with *F. graminearum*. At 48 h post inoculation, infected spikes were collected, immediately frozen in liquid nitrogen, and stored at −80 °C.

### 4.3. RNA Extraction, Sequencing, and In Silico Analysis

The TRI reagent method was used to isolate total RNA from ultra-frozen spikes. The frozen tissues were placed in a mortar containing liquid nitrogen and ground with a pestle to obtain fine powder. Ground tissue samples were vortexed with TRI reagent solution at 10 °C for 1 h, and the lysates were centrifuged. The supernatant was transferred to a tube with 5PRIME Phase Lock Gel Heavy, which was used to remove impurities from the aqueous phase. RNA samples were treated with RNase-free DNase (Qiagen, Hilden, Germany) and cleaned up using the RNeasy Mini Kit (Qiagen, Hilden, Germany) according to the manufacturer’s instructions [30]. RNA was quantified and quality-checked prior to sequencing with the use of a BioAnalyzer 2100 (Agilent Technologies, Santa Clara, CA, USA).

The mRNA sequence libraries were prepared using the TruSeq Stranded mRNA Sample Prep Kit (RS-122; Illumina, San Diego, CA, USA). Sequencing was performed using an Illumina NovaSeq 6000 sequencer (2 × 150 bp paired-end runs) (Macrogen, Seoul, Republic of Korea). The quality of sequenced 2 × 150 nt raw paired-end reads was checked using FastQC [79]. Raw paired-end reads were processed with Trimmomatic (v0.38) [80] to remove adapter sequences, low-quality nucleotides (Q = 20), and standardize read lengths. Adapter removal was performed using the TruSeq3-PE-2 library (parameters: ILLUMINACLIP:TruSeq3-PE-2.fa:2:30:10). The reads were subsequently hard-cropped to a maximum length of 130 bp (CROP:130), and any reads shorter than 130 bp were discarded (MINLEN:130) to ensure uniform read lengths for downstream analyses.

Processed reads were mapped to the *Triticum turgidum* reference genome (Svevo v1) using STAR (v2.7.1a) [81]. Gene annotation version v1.45 (Ensembl Plants) was used for transcriptome indexing and quantification. STAR was run with parameters recommended by the ENCODE consortium. Specifically, the maximum number of multiple alignments allowed for a read was set to 20 (outFilterMultimapNmax 20). The minimum and maximum intron lengths were set to 20 bp and 1 Mb, respectively, (alignIntronMin 20, alignIntronMax 1000000). Spurious splice junctions were filtered using the --outFilterType BySJout option.

Gene abundance quantification was performed simultaneously during alignment using the STAR –quantMode GeneCounts option. StringTie software v1.3.5 [82] was applied to annotate and estimate the expression of genes and transcripts. Counts per gene and per transcript were computed using the prepDE Python script (prepDE.py3) provided by the StringTie. The DEGs analysis process was conducted using DESeq2 with the parameters: *q*-value < 0.05 and |log2(FC)| ≥ 1. The unmapped sequences, to the reference genomes *T. turgidum* (Svevo.v1) and *F. graminearum* [45], were imported into OmicsBox v1.4 (available at https://www.biobam.com/ accessed on 1 February 2021) to perform mapping to *D. hansenii*. InterPro protein signatures and domain hits were obtained using InterProScan 5.60-92.0.

The DEGs of *T. turgidum* were subjected to functional classification using the DAVID gene functional classification tool (DAVID 2021; [41]). Enrichment analyses were performed with ShinyGo 0.82 [83] and STRING v12 [84]. Potential transcription factors (TFs), transcriptional regulators (TRs), and protein kinases (PKs) were identified with the iTAK program (v1.6 with database updated to 18.12) [85]. Data visualization and graphing were performed with SRplot [86].

### 4.4. Quantitative Real-Time PCR (RT-qPCR) Analysis

Five DEGs were selected for the validation of RNA-seq data using RT-qPCR. The selection of DEGs for validation was based on results from the DAVID Gene Functional Classification Tool and Gene Ontology (GO) analyses. The same RNA samples (from three biological replicates) that were used for sequencing were submitted to RT-qPCR. RNA (3.5 µg) was reverse transcribed using an oligo dT primer and the RETROscript kit (Ambion, Austin, TX, USA) at 42 °C for 2 h. The expression of *T. turgidum* selected genes was determined with the use of specific primers (Table S10). Quantitative RT-PCR was performed with a reagent kit SYBR Select Master Mix (Thermo Fisher Scientific, Waltham, MA, USA) in a reaction volume of 20 μL with 200 nM of each primer and 4 μL of diluted cDNA (10 times). A melt curve analysis was performed in the last step. The relative expression of abundance of wheat transcripts was normalized based on the Ct values of two reference genes (glyceraldehyde-3-phosphate dehydrogenase (GAPDH) and hn-RNP-Q), as calculated by Vandesompele et al. [87]. Three independent biological and two technical replicates were performed for each experiment. For data processing, normalized reporter values (Rn) were analyzed using LinRegPCR software v11.1 [88]. The significance of differences between the two samples (Figure 6) was evaluated using an unequal variance *t*-test in GraphPad Prism software (version 6.07). Differences were considered statistically significant at *p* < 0.05.

## 5. Conclusions

Priming wheat seedlings with *D. hansenii* significantly reprograms the expression of genes induced by *F. graminearum* inoculation, revealing multiple layers of plant defense activation. Notably, transcripts encoding proteins involved in cell-wall metabolism, including biosynthesis and modification enzymes, were altered, suggesting that *D. hansenii* may strengthen the plant’s primary physical barrier against pathogens. In addition, genes associated with signal transduction and detoxification of fungal toxins were differentially expressed, indicating that the yeast may modulate both recognition and response pathways in the host. Furthermore, the *D. hansenii* J1 strain, which harbors genes encoding killer proteins, may directly inhibit pathogen growth. Collectively, these results provide new insights into the molecular mechanisms by which *D. hansenii* enhances wheat resistance to *F. graminearum*. However, to fully understand the impact of *D. hansenii* use on the plant defense mechanism, further research is necessary.

## Data Availability

The original contributions presented in this study are included in the article and/or the Supplementary Materials. The data have been deposited in the ENA database under accession number PRJEB55781. Further inquiries may be directed to the corresponding author upon reasonable request.

## Supplementary Materials

The following supporting information can be downloaded at: https://www.mdpi.com/article/10.3390/ijms27010457/s1.

## References

[1] Huang S., Sirikhachornkit A., Su X., Faris J., Gill B., Haselkorn R., Gornicki P. (2002). Genes encoding plastid acetyl-CoA carboxylase and 3-phosphoglycerate kinase of the *Triticum/Aegilops* complex and the evolutionary history of polyploid wheat. Proc. Natl. Acad. Sci. USA.

[2] FAO (2023). FAOSTAT: Production: Crop and Livestock Products. https://www.fao.org/faostat/en/#data/QCL.

[3] Blanco A. (2024). Structure and Trends of Worldwide Research on Durum Wheat by Bibliographic Mapping. Int. J. Plant Biol..

[4] Gai X.T., Xuan Y.H., Gao Z.G. (2017). Diversity and pathogenicity of *Fusarium graminearum* species complex from maize stalk and ear rot strains in northeast China. Plant Pathol..

[5] O’Donnell K. (2000). Molecular phylogeny of the *Nectria haematococca*-*Fusarium solani* species complex. Mycologia.

[6] O’Donnell K., Sutton D.A., Fothergill A., McCarthy D., Rinaldi M.G., Brandt M.E., Zhang N., Geiser D.M. (2008). Molecular Phylogenetic Diversity, Multilocus Haplotype Nomenclature, and In Vitro Antifungal Resistance within the *Fusarium solani* Species Complex. J Clin. Microbiol..

[7] Starkey D.E., Ward T.J., Aoki T., Gale L.R., Kistler H.C., Geiser D.M., Suga H., Tóth B., Varga J., O’Donnell K. (2007). Global molecular surveillance reveals novel Fusarium head blight species and trichothecene toxin diversity. Fungal Genet. Biol..

[8] Yli-Mattila T., Gagkaeva T., Ward T.J., Aoki T., Kistler H.C., O’Donnell K. (2009). A novel Asian clade within the *Fusarium graminearum* species complex includes a newly discovered cereal head blight pathogen from the Russian Far East. Mycologia.

[9] Sarver B.A., Ward T.J., Gale L.R., Broz K., Kistler H.C., Aoki T., Nicholson P., Carter J., O’Donnell K. (2011). Novel Fusarium head blight pathogens from Nepal and Louisiana revealed by multilocus genealogical concordance. Fungal Genet. Biol..

[10] Garofalo M., Payros D., Penary M., Oswald E., Nougayrède J.-P., Oswald I.P. (2023). A novel toxic effect of foodborne trichothecenes: The exacerbation of genotoxicity. Environ. Pollut..

[11] Simsek S., Ovando-Martínez M., Ozsisli B., Whitney K., Ohm J.-B. (2013). Occurrence of Deoxynivalenol and Deoxynivalenol-3-glucoside in Hard Red Spring Wheat Grown in the USA. Toxins.

[12] McMullen M., Bergstrom G., De Wolf E., Dill-Macky R., Hershman D., Shaner G., Van Sanford D. (2012). A Unified Effort to Fight an Enemy of Wheat and Barley: Fusarium Head Blight. Plant Dis..

[13] Franco M.F., Lori G.A., Cendoya G., Alonso M.P., Panelo J.S., Malbrán I., Mirabella N.E., Pontaroli A.C. (2021). Spike architecture traits associated with type II resistance to fusarium head blight in bread wheat. Euphytica.

[14] Felici L., Francesconi S., Sestili F., Balestra G.M. (2024). Physiological and morphological traits associated with Fusarium head blight response in a flavonoid-rich durum wheat genotype. J. Plant Pathol..

[15] Lionetti V., Giancaspro A., Fabri E., Giove S.L., Reem N., A Zabotina O., Blanco A., Gadaleta A., Bellincampi D. (2015). Cell wall traits as potential resources to improve resistance of durum wheat against *Fusarium graminearum*. BMC Plant Biol..

[16] Giancaspro A., Lionetti V., Giove S.L., Zito D., Fabri E., Reem N., Zabotina O.A., De Angelis E., Monaci L., Bellincampi D. (2018). Cell wall features transferred from common into durum wheat to improve Fusarium Head Blight resistance. Plant Sci..

[17] Chen C., Guo Q., He Q., Tian Z., Hao W., Shan X., Lu J., Barkla B.J., Ma C., Si H. (2023). Comparative transcriptomic analysis of wheat cultivars differing in their resistance to Fusarium head blight infection during grain-filling stages reveals unique defense mechanisms at play. BMC Plant Biol..

[18] Ribichich K.F., Lopez S.E., Vegetti A.C. (2000). Histopathological Spikelet Changes Produced by *Fusarium graminearum* in Susceptible and Resistant Wheat Cultivars. Plant Dis..

[19] Prat N., Buerstmayr M., Steiner B., Robert O., Buerstmayr H. (2014). Current knowledge on resistance to Fusarium head blight in tetraploid wheat. Mol. Breed..

[20] Haile J.K., Sertse D., N’dIaye A., Klymiuk V., Wiebe K., Ruan Y., Chawla H.S., Henriquez M.-A., Wang L., Kutcher H.R. (2023). Multi-locus genome-wide association studies reveal the genetic architecture of Fusarium head blight resistance in durum wheat. Front. Plant Sci..

[21] Miedaner T., Longin C.F.H. (2014). Genetic variation for resistance to Fusarium head blight in winter durum material. Crop. Pasture Sci..

[22] Miedaner T., Sieber A., Desaint H., Buerstmayr H., Longin C.F.H., Würschum T. (2017). The potential of genomic-assisted breeding to improve Fusarium head blight resistance in winter durum wheat. Plant Breed..

[23] Buerstmayr M., Steiner B., Buerstmayr H. (2020). Breeding for Fusarium head blight resistance in wheat—Progress and challenges. Plant Breed..

[24] Fabre F., Rocher F., Alouane T., Langin T., Bonhomme L. (2020). Searching for FHB Resistances in Bread Wheat: Susceptibility at the Crossroad. Front. Plant Sci..

[25] Buerstmayr M., Huber K., Heckmann J., Steiner B., Nelson J.C., Buerstmayr H. (2012). Mapping of QTL for Fusarium head blight resistance and morphological and developmental traits in three backcross populations derived from *Triticum dicoccum* × *Triticum durum*. Theor. Appl. Genet..

[26] Zhang Z., Belcram H., Gornicki P., Charles M., Just J., Huneau C., Magdelenat G., Couloux A., Samain S., Gill B.S. (2011). Duplication and partitioning in evolution and function of homoeologous *Q* loci governing domestication characters in polyploid wheat. Proc. Natl. Acad. Sci. USA.

[27] Kirana R.P., Gaurav K., Arora S., Wiesenberger G., Doppler M., Michel S., Zimmerl S., Matic M., Eze C.E., Kumar M. (2023). Identification of a UDP-glucosyltransferase conferring deoxynivalenol resistance in *Aegilops tauschii* and wheat. Plant Biotechnol. J..

[28] Kirana R.P., Michel S., Moreno-Amores J., Prat N., Lemmens M., Buerstmayr M., Buerstmayr H., Steiner B. (2023). Pyramiding Fusarium head blight resistance QTL from *T. aestivum*, *T. dicoccum* and *T. dicoccoides* in durum wheat. Theor. Appl. Genet..

[29] Ding L., Xu H., Yi H., Yang L., Kong Z., Zhang L., Xue S., Jia H., Ma Z. (2011). Resistance to Hemi-Biotrophic *F. graminearum* Infection Is Associated with Coordinated and Ordered Expression of Diverse Defense Signaling Pathways. PLoS ONE.

[30] Qi P.-F., Balcerzak M., Rocheleau H., Leung W., Wei Y.-M., Zheng Y.-L., Ouellet T. (2016). Jasmonic acid and abscisic acid play important roles in host–pathogen interaction between *Fusarium graminearum* and wheat during the early stages of fusarium head blight. Physiol. Mol. Plant Pathol..

[31] Brauer E.K., Rocheleau H., Balcerzak M., Pan Y., Fauteux F., Liu Z., Wang L., Zheng W., Ouellet T. (2019). Transcriptional and hormonal profiling of *Fusarium graminearum*-infected wheat reveals an association between auxin and susceptibility. Physiol. Mol. Plant Pathol..

[32] Klocke B., Sommerfeldt N., Wagner C., Schwarz J., Baumecker M., Ellmer F., Jacobi A., Matschiner K., Petersen J., Wehling P. (2023). Disease threshold-based fungicide applications: Potential of multi-disease resistance in winter wheat cultivars in Germany. Eur. J. Plant Pathol..

[33] Talas F., McDonald B.A. (2015). Genome-wide analysis of *Fusarium graminearum* field populations reveals hotspots of recombination. BMC Genom..

[34] Matelionienė N., Žvirdauskienė R., Kadžienė G., Zavtrikovienė E., Supronienė S. (2024). In Vitro Sensitivity Test of *Fusarium* Species from Weeds and Non-Gramineous Plants to Triazole Fungicides. Pathogens.

[35] Ivic D., Sever Z., Kuzmanovska B. (2011). In vitro sensitivity of *Fusarium graminearum*, *F. avenaceum* and *F. verticillioides* to carbendazim, tebuconazole, flutriafol, metconazole and prochloraz. Pestic. Fitomedicina.

[36] (2009). Directive 2009/128/EC of the European Parliament and of the Council of 21 October 2009 establishing a framework for Community action to achieve the sustainable use of pesticides. Off. J. Eur. Union.

[37] Fröhlich-Wyder M., Arias-Roth E., Jakob E. (2019). Cheese yeasts. Yeast.

[38] Koutsoumanis K., Allende A., Alvarez-Ordóñez A., Bolton D., Bover-Cid S., Chemaly M., Davies R., De Cesare A., Hilbert F., Lindqvist R. (2020). Update of the list of QPS-recommended biological agents intentionally added to food or feed as notified to EFSA 12: Suitability of taxonomic units notified to EFSA until March 2020. EFSA J..

[39] Grzegorczyk M., Żarowska B., Restuccia C., Cirvilleri G. (2017). Postharvest biocontrol ability of killer yeasts against *Monilinia fructigena* and *Monilinia fructicola* on stone fruit. Food Microbiol..

[40] Wachowska U., Sulyok M., Wiwart M., Suchowilska E., Kandler W., Krska R. (2022). The application of antagonistic yeasts and bacteria: An assessment of in vivo and under field conditions pattern of *Fusarium* mycotoxins in winter wheat grain. Food Control.

[41] Huang D.W., Sherman B.T., Tan Q., Collins J.R., Alvord W.G., Roayaei J., Stephens R., Baseler M.W., Lane H.C., Lempicki R.A. (2007). The DAVID Gene Functional Classification Tool: A novel biological module-centric algorithm to functionally analyze large gene lists. Genome Biol..

[42] Medina-Córdova N., López-Aguilar R., Ascencio F., Castellanos T., Campa-Córdova A.I., Angulo C. (2016). Biocontrol activity of the marine yeast *Debaryomyces hansenii* against phytopathogenic fungi and its ability to inhibit mycotoxins production in maize grain (*Zea mays* L.). Biol. Control.

[43] Rivas-Garcia T., Murillo-Amador B., Reyes-Pérez J.J., Chiquito-Contreras R.G., Preciado-Rangel P., Ávila-Quezada G.D., Lara-Capistran L., Hernandez-Montiel L.G. (2022). *Debaryomyces hansenii*, *Stenotrophomonas rhizophila*, and Ulvan as Biocontrol Agents of Fruit Rot Disease in Muskmelon (*Cucumis melo* L.). Plants.

[44] Payne C., Bruce A. (2001). The Yeast *Debaryomyces hansenii* as a Short-Term Biological Control Agent against Fungal Spoilage of Sawn *Pinus sylvestris* Timber. Biol. Control.

[45] Wachowska U., Pluskota W., Jastrzębski J.P., Głowacka K., Szablewska-Stuper K., Balcerzak M. (2023). A method for reducing the concentrations of *Fusarium graminearum* trichothecenes in durum wheat grain with the use of *Debaryomyces hansenii*. Int. J. Food Microbiol..

[46] Giedrojć W., Wachowska U. (2025). Mycobiome and Pathogenic *Fusarium* Fungi in the Rhizosphere of Durum Wheat After Seed Dressing with *Debaryomyces hansenii*. Agriculture.

[47] Sevillano-Caño J., García M.J., Córdoba-Galván C., Luque-Cruz C., Agustí-Brisach C., Lucena C., Ramos J., Pérez-Vicente R., Romera F.J. (2024). Exploring the Role of *Debaryomyces hansenii* as Biofertilizer in Iron-Deficient Environments to Enhance Plant Nutrition and Crop Production Sustainability. Int. J. Mol. Sci..

[48] Núñez-Cano J., Ruiz-Castilla F.J., Ramos J., Romera F.J., Lucena C. (2025). *Debaryomyces hansenii* Enhances Growth, Nutrient Uptake, and Yield in Rice Plants (*Oryza sativa* L.) Cultivated in Calcareous Soil. Agronomy.

[49] Narusaka M., Minami T., Iwabuchi C., Hamasaki T., Takasaki S., Kawamura K., Narusaka Y. (2015). Yeast Cell Wall Extract Induces Disease Resistance against Bacterial and Fungal Pathogens in *Arabidopsis thaliana* and *Brassica* Crop. PLoS ONE.

[50] Yaguchi T., Kinami T., Ishida T., Yasuhara T., Takahashi K., Matsuura H. (2017). Induction of plant disease resistance upon treatment with yeast cell wall extract. Biosci. Biotechnol. Biochem..

[51] Gao X., Li F., Sun Y., Jiang J., Tian X., Li Q., Duan K., Lin J., Liu H., Wang Q. (2024). Basal defense is enhanced in a wheat cultivar resistant to Fusarium head blight. J. Integr. Agric..

[52] del Carmen Orozco-Mosqueda M., Glick B.R., Santoyo G. (2020). ACC deaminase in plant growth-promoting bacteria (PGPB): An efficient mechanism to counter salt stress in crops. Microbiol. Res..

[53] Luo J., Zhou J.-J., Zhang J.-Z. (2018). Aux/IAA Gene Family in Plants: Molecular Structure, Regulation, and Function. Int. J. Mol. Sci..

[54] Chen X., Steed A., Travella S., Keller B., Nicholson P. (2009). *Fusarium graminearum* exploits ethylene signalling to colonize dicotyledonous and monocotyledonous plants. New Phytol..

[55] Su P., Zhao L., Li W., Zhao J., Yan J., Ma X., Li A., Wang H., Kong L. (2021). Integrated metabolo-transcriptomics and functional characterization reveals that the wheat auxin receptor TIR1 negatively regulates defense against *Fusarium graminearum*. J. Integr. Plant Biol..

[56] Pan Y., Liu Z., Rocheleau H., Fauteux F., Wang Y., McCartney C., Ouellet T. (2018). Transcriptome dynamics associated with resistance and susceptibility against fusarium head blight in four wheat genotypes. BMC Genom..

[57] Rocher F., Dou S., Philippe G., Martin M.-L., Label P., Langin T., Bonhomme L. (2024). Integrative systems biology of wheat susceptibility to *Fusarium graminearum* uncovers a conserved gene regulatory network and identifies master regulators targeted by fungal core effectors. BMC Biol..

[58] Thapa G., Gunupuru L.R., Hehir J.G., Kahla A., Mullins E., Doohan F.M. (2018). A Pathogen-Responsive Leucine Rich Receptor Like Kinase Contributes to *Fusarium* Resistance in Cereals. Front. Plant Sci..

[59] Yan J., Su P., Meng X., Liu P. (2023). Phylogeny of the plant receptor-like kinase (RLK) gene family and expression analysis of wheat RLK genes in response to biotic and abiotic stresses. BMC Genom..

[60] Passardi F., Penel C., Dunand C. (2004). Performing the paradoxical: How plant peroxidases modify the cell wall. Trends Plant Sci..

[61] Paniagua C., Bilkova A., Jackson P., Dabravolski S., Riber W., Didi V., Houser J., Gigli-Bisceglia N., Wimmerova M., Budínská E. (2017). Dirigent proteins in plants: Modulating cell wall metabolism during abiotic and biotic stress exposure. J. Exp. Bot..

[62] Sun Z., Zhou Y., Hu Y., Jiang N., Hu S., Li L., Li T. (2022). Identification of Wheat LACCASEs in Response to *Fusarium graminearum* as Potential Deoxynivalenol Trappers. Front. Plant Sci..

[63] Ursache R., Teixeira C.D.J.V., Tendon V.D., Gully K., De Bellis D., Schmid-Siegert E., Andersen T.G., Shekhar V., Calderon S., Pradervand S. (2021). GDSL-domain proteins have key roles in suberin polymerization and degradation. Nat. Plants.

[64] Basheer J., Vadovič P., Šamajová O., Melicher P., Komis G., Křenek P., Králová M., Pechan T., Ovečka M., Takáč T. (2022). Knockout of MITOGEN-ACTIVATED PROTEIN KINASE 3 causes barley root resistance against *Fusarium graminearum*. Plant Physiol..

[65] Eldakak M., Das A., Zhuang Y., Rohila J.S., Glover K., Yen Y. (2018). A Quantitative Proteomics View on the Function of Qfhb1, a Major QTL for Fusarium Head Blight Resistance in Wheat. Pathogens.

[66] Schweiger W., Steiner B., Vautrin S., Nussbaumer T., Siegwart G., Zamini M., Jungreithmeier F., Gratl V., Lemmens M., Mayer K.F.X. (2016). Suppressed recombination and unique candidate genes in the divergent haplotype encoding Fhb1, a major Fusarium head blight resistance locus in wheat. Theor. Appl. Genet..

[67] Bischoff V., Nita S., Neumetzler L., Schindelasch D., Urbain A., Eshed R., Persson S., Delmer D., Scheible W.-R. (2010). TRICHOME BIREFRINGENCE and Its Homolog AT5G01360 Encode Plant-Specific DUF231 Proteins Required for Cellulose Biosynthesis in Arabidopsis. Plant Physiol..

[68] Huang Y., Li L., Smith K.P., Muehlbauer G.J. (2016). Differential transcriptomic responses to *Fusarium graminearum* infection in two barley quantitative trait loci associated with Fusarium head blight resistance. BMC Genom..

[69] Gille S., Pauly M. (2012). O-Acetylation of Plant Cell Wall Polysaccharides. Front. Plant Sci..

[70] Lin S., Miao Y., Huang H., Zhang Y., Huang L., Cao J. (2022). Arabinogalactan Proteins: Focus on the Role in Cellulose Synthesis and Deposition during Plant Cell Wall Biogenesis. Int. J. Mol. Sci..

[71] Medina-Córdova N., Rosales-Mendoza S., Hernández-Montiel L.G., Angulo C. (2018). The potential use of *Debaryomyces hansenii* for the biological control of pathogenic fungi in food. Biol. Control.

[72] Hernandez-Montiel L.G., Gutierrez-Perez E.D., Murillo-Amador B., Vero S., Chiquito-Contreras R.G., Rincon-Enriquez G. (2018). Mechanisms employed by *Debaryomyces hansenii* in biological control of anthracnose disease on papaya fruit. Postharvest Biol. Technol..

[73] Li Z., Chen Y., Liu D., Zhao N., Cheng H., Ren H., Guo T., Niu H., Zhuang W., Wu J. (2015). Involvement of glycolysis/gluconeogenesis and signaling regulatory pathways in *Saccharomyces cerevisiae* biofilms during fermentation. Front. Microbiol..

[74] Czarnecka M., Żarowska B., Połomska X., Restuccia C., Cirvilleri G. (2019). Role of biocontrol yeasts *Debaryomyces hansenii* and *Wickerhamomyces anomalus* in plants’ defence mechanisms against *Monilinia fructicola* in apple fruits. Food Microbiol..

[75] Lukša J., Podoliankaitė M., Vepštaitė I., Strazdaitė-Žielienė Ž., Urbonavičius J., Servienė E. (2015). Yeast β-1,6-Glucan Is a Primary Target for the *Saccharomyces cerevisiae* K2 Toxin. Eukaryot. Cell.

[76] Billerbeck S., Walker R.S., Pretorius I.S. (2024). Killer yeasts: Expanding frontiers in the age of synthetic biology. Trends Biotechnol..

[77] Liu G.-L., Chi Z., Wang G.-Y., Wang Z.-P., Li Y., Chi Z.-M. (2015). Yeast killer toxins, molecular mechanisms of their action and their applications. Crit. Rev. Biotechnol..

[78] Meier U., Schwartz M.D. (2003). Phenological Growth Stages. Phenology: An Integrative Environmental Science; Tasks for Vegetation Science.

[79] Andrews S. (2010). FastQC: A Quality Control Tool for High Throughput Sequence Data. http://www.bioinformatics.babraham.ac.uk/projects/fastqc/.

[80] Bolger A.M., Lohse M., Usadel B. (2014). Trimmomatic: A flexible trimmer for Illumina sequence data. Bioinformatics.

[81] Dobin A., Davis C.A., Schlesinger F., Drenkow J., Zaleski C., Jha S., Batut P., Chaisson M., Gingeras T.R. (2013). STAR: Ultrafast universal RNA-seq aligner. Bioinformatics.

[82] Pertea M., Pertea G.M., Antonescu C.M., Chang T.-C., Mendell J.T., Salzberg S.L. (2015). StringTie enables improved reconstruction of a transcriptome from RNA-seq reads. Nat. Biotechnol..

[83] Ge S.X., Jung D., Yao R. (2020). ShinyGO: A graphical gene-set enrichment tool for animals and plants. Bioinformatics.

[84] Szklarczyk D., Kirsch R., Koutrouli M., Nastou K., Mehryary F., Hachilif R., Gable A.L., Fang T., Doncheva N.T., Pyysalo S. (2023). The STRING database in 2023: Protein–protein association networks and functional enrichment analyses for any sequenced genome of interest. Nucleic Acids Res..

[85] Zheng Y., Jiao C., Sun H., Rosli H.G., Pombo M.A., Zhang P., Banf M., Dai X., Martin G.B., Giovannoni J.J. (2016). iTAK: A Program for Genome-wide Prediction and Classification of Plant Transcription Factors, Transcriptional Regulators, and Protein Kinases. Mol. Plant.

[86] Tang D., Chen M., Huang X., Zhang G., Zeng L., Zhang G., Wu S., Wang Y. (2023). SRplot: A free online platform for data visualization and graphing. PLoS ONE.

[87] Vandesompele J., De Preter K., Pattyn F., Poppe B., Van Roy N., De Paepe A., Speleman F. (2002). Accurate normalization of real-time quantitative RT-PCR data by geometric averaging of multiple internal control genes. Genome Biol..

[88] Ruijter J.M., Ramakers C., Hoogaars W.M.H., Karlen Y., Bakker O., van den Hoff M.J.B., Moorman A.F.M. (2009). Amplification efficiency: Linking baseline and bias in the analysis of quantitative PCR data. Nucleic Acids Res..

