# Supplementary material for: Transcriptional Response of Durum Wheat During Interaction with Debaryomyces hansenii and Fusarium graminearum
Source: Int J Mol Sci. 2026 Jan 1;27(1):457. doi: 10.3390/ijms27010457 (PMC12786629; doi:10.3390/ijms27010457)
Supplement: Supplementary file 1 [file ijms-27-00457-s001.zip › Figure S1.pdf]

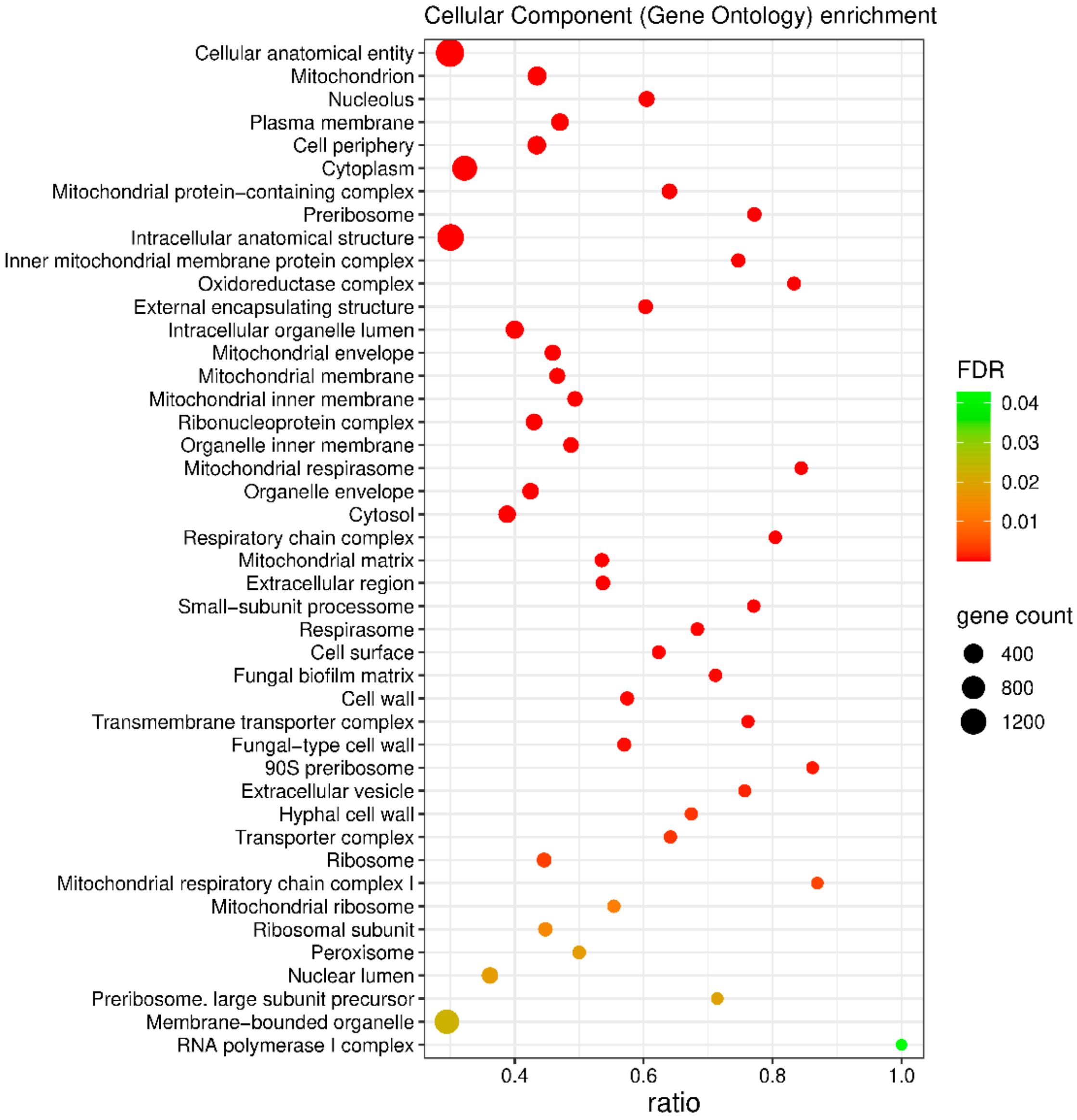

Figure S1. GO analysis of mapped *D. hansenii* genes with cellular components enrichment.; point size denotes gene count, color denotes the p-value and the x-axis denotes the gene ratio.
