# Supplementary material for: Transcriptional Response of Durum Wheat During Interaction with Debaryomyces hansenii and Fusarium graminearum
Source: Int J Mol Sci. 2026 Jan 1;27(1):457. doi: 10.3390/ijms27010457 (PMC12786629; doi:10.3390/ijms27010457)
Supplement: Supplementary file 1 [file ijms-27-00457-s001.zip › Figure S2.pdf]

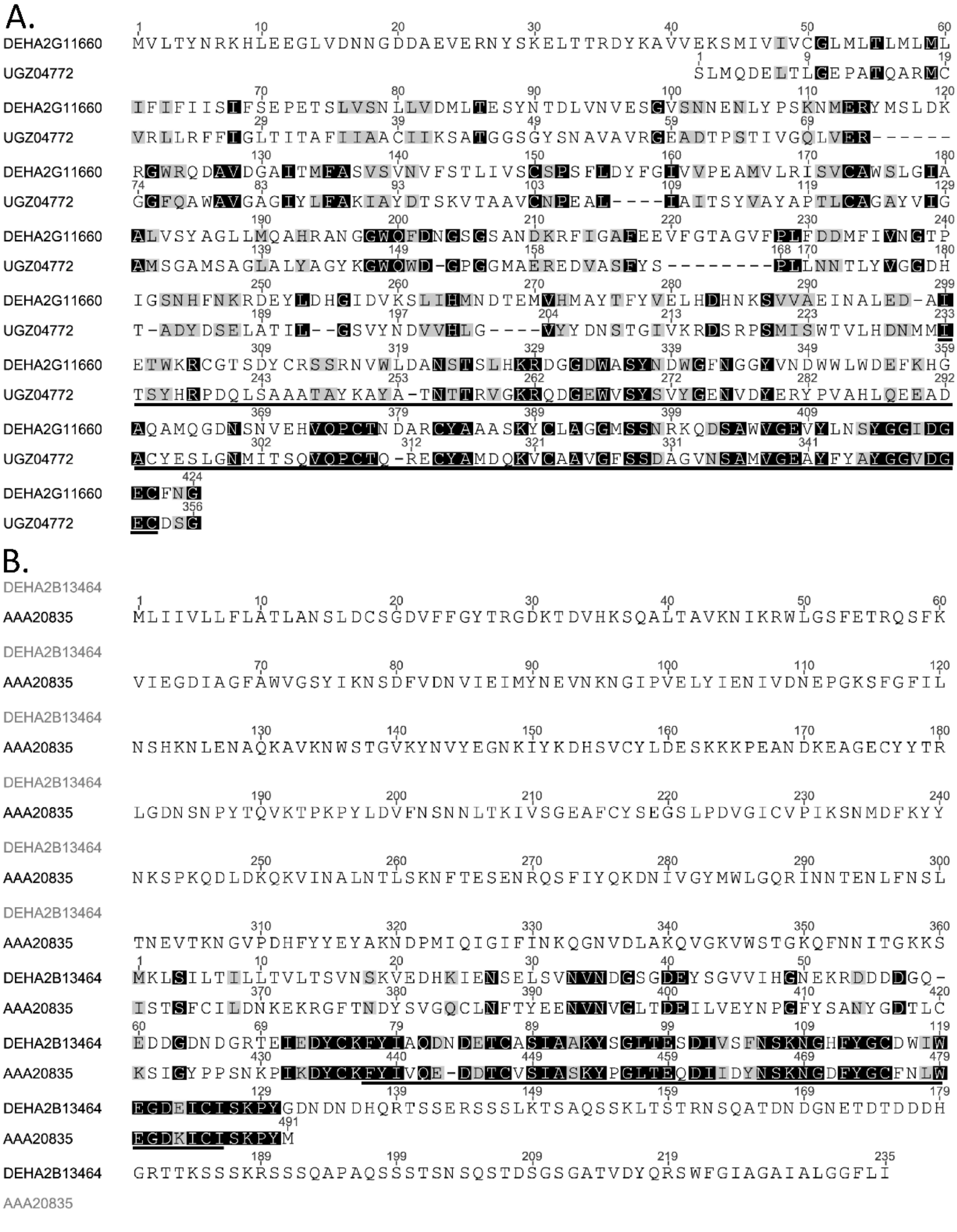

Figure S2. Alignment of amino acid sequences of potential *D. hansenii* killer toxin encoded by DEHA2G11660 (A) and DEHA2B13464 (B) with amino acid sequences of killer toxin from *Saccharomyces cerevisiae* (A, UGZ04772) and protein *Millerozyma acacia* similar to *Kluyveromyces lactis* killer toxin (B, AAA20835). The C-terminal domains of PF17276 (A) and PF01476 (B) are underlined, and identical amino acids are marked with a black background.
