# Supplementary material for: Transcriptional Response of Durum Wheat During Interaction with Debaryomyces hansenii and Fusarium graminearum
Source: Int J Mol Sci. 2026 Jan 1;27(1):457. doi: 10.3390/ijms27010457 (PMC12786629; doi:10.3390/ijms27010457)
Supplement: Supplementary file 1 [file ijms-27-00457-s001.zip › Table S1.pdf]

Table S1. Summary of RNA-Seq reads from *Triticum turgidum* spp. *durum* mapped to the genome (*T. turgidum* annotation version Svevo.v1). The table summarizes total, trimmed, and uniquely mapped reads, as well as reads aligned to annotated genes, with percentages calculated relative to the corresponding read category.

Treatment	Rep number	Total reads	Trimmed reads		Uniquely mapped reads		Reads aligned to genes	
		(M)	(M)	(%)	(M)	(%)	(M)	(%)
Dh/Fg	1	62.7	53.9	85.96	49.0	90.82	34.1	69.76
Dh/Fg	2	71.4	54.6	76.47	49.0	89.67	39.2	80.02
Dh/Fg	3	87.1	65.8	75.55	59.4	90.27	46.6	78.46
Mean (± SE)		73.7 (±7.139)	58.1 (±3.855)	79.33 (±3.327)	52.4 (±3.480)	90.25 (±0.332)	40.0 (±3.617)	76.08 (±3.192)
Fg	1	70.4	55.8	79.26	50.2	90.02	39.9	79.35
Fg	2	64.8	54.9	84.72	48.7	88.74	37.5	77.03
Fg	3	55.4	47.0	84.84	40.7	86.62	31.8	78.22
Mean (± SE)		63.5 (±4.376)	52.6 (±2.795)	82.94 (±1.840)	46.6 (±2.953)	88.46 (±0.991)	36.4 (±2.380)	78.20 (±0.670)

Dh – *Debaryomyces hansenii*; Fg – *Fusarium graminearum*; SE – standard error; M – 10^6
