# Supplementary material for: Transcriptional Response of Durum Wheat During Interaction with Debaryomyces hansenii and Fusarium graminearum
Source: Int J Mol Sci. 2026 Jan 1;27(1):457. doi: 10.3390/ijms27010457 (PMC12786629; doi:10.3390/ijms27010457)
Supplement: Supplementary file 1 [file ijms-27-00457-s001.zip › Table S10.pdf]

Table S10. Primers used in this study for expression analysis of wheat genes.

Gene name	Sequence (5' → 3') of forward (F) and reverse (R) primers	Efficiency
DIR (TRITD2Av1G273910)	F: GCCTCACCTCTGCATTTCTAGCT	1,844
	R: CGTGCATGTAGAAGTGGAGATGTT	
GDSL (TRITD7Av1G231170)	F: CGCGTTCTTCGGGAACAA	1,841
	R: GAAGTTGCCCGGGACGAT	
BCP (TRITD6Bv1G185120)	F: GCCGCCACCAGCTTCAC	1,835
	R: TGGCGTAGTTGAACACGAGACT	
POD (TRITD7Bv1G190550)	F: AATTGCACCACACCCACTGA	1,851
	R: AAGAGTCCCTGGCGCTTGA	
ERF (TRITD2Bv1G206010)	F: TAACGATGATCAGGGCCCTTT	1,795
	R: GAAGAACATGGGCTGCTGGTA	
GAPDH	F: AACTGTTCATGCCATCACTGCCAC	1,817
	R: AGGACATACCAGTGAGCTTGCCAT	
hn-RNP-Q	F: TCACCTTCGCCAAGCTCAGAACTA	1,837
	R: AGTTGAACTTGCCCGAAACATGCC	
