# Supplementary material for: Transcriptional Response of Durum Wheat During Interaction with Debaryomyces hansenii and Fusarium graminearum
Source: Int J Mol Sci. 2026 Jan 1;27(1):457. doi: 10.3390/ijms27010457 (PMC12786629; doi:10.3390/ijms27010457)
Supplement: Supplementary file 1 [file ijms-27-00457-s001.zip › Table S2.pdf]

Table S2. Results of the principal component analysis (PCA) of RNA-Seq samples. PC1 and PC2 represent the first and second principal components, respectively.

Variable	Rep number	PC1	PC2
Dh/Fg	1	-22,45	7,24
Dh/Fg	2	-6,08	8,67
Dh/Fg	3	17,17	5,19
Fg	1	19,78	0,21
Fg	2	2,37	-8,19
Fg	3	-10,79	-13,12

Dh – *Debaryomyces hansenii*; Fg – *Fusarium graminearum*
