# Supplementary material for: Transcriptional Response of Durum Wheat During Interaction with Debaryomyces hansenii and Fusarium graminearum
Source: Int J Mol Sci. 2026 Jan 1;27(1):457. doi: 10.3390/ijms27010457 (PMC12786629; doi:10.3390/ijms27010457)
Supplement: Supplementary file 1 [file ijms-27-00457-s001.zip › Table S8.pdf]

Table S8. KEGG categories of significantly enriched *D. hansenii* mRNA transcripts.

KEGG pathway ID	KEGG pathway name	KEGG main category	Exact p-value	Adjusted p-value	number of genes
1100	Metabolic pathways	Metabolism	6,03E-24	5,97E-22	321
1110	Biosynthesis of secondary metabolites	Metabolism	4,75E-20	2,35E-18	150
1230	Biosynthesis of amino acids	Metabolism	6,78E-13	2,24E-11	70
1200	Carbon metabolism	Metabolism	3,51E-11	7,38E-10	62
190	Oxidative phosphorylation	Metabolism	3,73E-11	7,38E-10	61
3008	Ribosome biogenesis in eukaryotes	Genetic Information Processing	9,41E-09	1,55E-07	48
20	Citrate cycle (TCA cycle)	Metabolism	1,43E-08	2,02E-07	22
3020	RNA polymerase	Genetic Information Processing	3,14E-07	3,89E-06	23
1210	2-Oxocarboxylic acid metabolism	Metabolism	2,51E-06	2,69E-05	23
620	Pyruvate metabolism	Metabolism	2,72E-06	2,69E-05	24
230	Purine metabolism	Metabolism	3,13E-06	2,81E-05	50
260	Glycine, serine and threonine metabolism	Metabolism	3,09E-05	2,55E-04	22
240	Pyrimidine metabolism	Metabolism	3,34E-05	2,55E-04	39
30	Pentose phosphate pathway	Metabolism	1,41E-04	9,56E-04	16
10	Glycolysis / Gluconeogenesis	Metabolism	1,45E-04	9,56E-04	27
4146	Peroxisome	Cellular Processes	1,79E-04	1,11E-03	25
330	Arginine and proline metabolism	Metabolism	2,37E-04	1,38E-03	22
3010	Ribosome	Genetic Information Processing	2,78E-04	1,53E-03	58
71	Fatty acid degradation	Metabolism	3,05E-04	1,53E-03	14
250	Alanine, aspartate and glutamate metabolism	Metabolism	3,08E-04	1,53E-03	18
300	Lysine biosynthesis	Metabolism	4,64E-04	2,19E-03	10
1212	Fatty acid metabolism	Metabolism	2,39E-03	1,08E-02	16
680	Methane metabolism	Metabolism	4,11E-03	1,77E-02	16
630	Glyoxylate and dicarboxylate metabolism	Metabolism	6,36E-03	2,62E-02	12

3050	Proteasome	Genetic Information Processing	6,84E-03	2,71E-02	19
280	Valine, leucine and isoleucine degradation	Metabolism	1,26E-02	4,81E-02	11
